# Supplementary material for: Cryptic Divergence of Rochia nilotica (Gastropoda: Tegulidae) from Chuuk Lagoon, Federated States of Micronesia, Revealed by Morphological and Mitochondrial Genome Analyses
Source: Animals (Basel). 2025 Dec 2;15(23):3471. doi: 10.3390/ani15233471 (PMC12691490; doi:10.3390/ani15233471)
Supplement: Supplementary file 1 [file animals-15-03471-s001.zip › animals-3985339-supplementary.pdf]

1. Supplementary Method

To strengthen the genetic comparison of the *Rochia* population from Chuuk Lagoon, mitochondrial COX1 and 16S rRNA gene sequences were additionally obtained from five individuals of *Rochia nilotica* collected in Weno Island (voucher numbers: HNIBRIV 18782–18786). These sequences were included in the *p*-distance analyses to verify intraspecific variation within the Chuuk population.

For COX1 amplification, a new primer set (RONCOF: 5′-ATGCGATGGCTATTTTCTACAAATCATAAAGA-CATTGG-3′ / RNCOR: 5′-TACACCTCAGGATGACCAAAAAACCA-3′) was specifically designed based on the complete mitochondrial genome generated in this study. Amplification of the 16S rRNA gene was performed using universal primers 16SAR (5′-CGCCTGTTTATCAAAAACAT-3′) and 16SBR (5′-ACGTGATCTGAGTTCAGACCG-3′). PCR reactions were conducted in a total volume of 25 μL containing 12.5 μL of 2× Invitrogen Master Mix, 0.5 μM of each primer, and template DNA.

PCR cycling conditions for COX1 consisted of an initial denaturation at 95 °C for 10 min, followed by 30 cycles of 95 °C for 30 s, 62 °C for 30 s, and 72 °C for 1 min, with a final extension at 72 °C for 10 min. For 16S rRNA, the annealing temperature was adjusted to 55 °C, and all other steps were identical to the COX1 protocol.

All successfully amplified products were sequenced bidirectionally and deposited in the NCBI GenBank database (see Table S1 for accession numbers). The sequences were aligned using MAFFT (v7.520), and pairwise genetic distances (*p*-distances) were calculated in MEGA 12 using the proportion of differing nucleotides. The resulting p-distance values are reported as percentages in Tables S2 and S3.

2. Supplementary Tables

Table S1. List of sequences used in the phylogenetic tree. Sequences based on complete mitochondrial genomes are indicated in bold.

Species (Voucher number)	NCBI Accession Number		Location
	COX1	16S rRNA	
Rochia nilotica (HNIBRIV 18781)	**PV929813**	**PV929813**	**Chuuk**
R. nilotica (HNIBRIV 18782)	PX578333	PX578451	Chuuk
R. nilotica (HNIBRIV 18783)	PX578334	PX578452	Chuuk
R. nilotica (HNIBRIV 18784)	PX578335	PX578453	Chuuk
R. nilotica (HNIBRIV 18785)	PX578336	PX578454	Chuuk
R. nilotica (HNIBRIV 18786)	PX578337	PX578455	Chuuk
R. nilotica	MK934681.1	-	French Polynesia: Moorea Island
R. nilotica	KT149313.1	-	French Polynesia: Moorea Island
R. nilotica	EU530151.1	HE800772.1	New Caledonia
Rochia maxima	EU530150.1	HE800771.1	Japan
Rochia conus	HE800628	HE800769.1	The Philippines: Panglao
Rochia virgata	**KY205709.1**	**KY205709.1**	**Aqaba, Jordan**
Tectus pyramis	NC_036068.1	NC_036068.1	China
Tectus fenestratus	EU530149	HE800770.1	New Caledonia
Tectus tentorium	EU530152	HE800773.1	Australia
Tegula brunnea	**NC_016954.1**	**NC_016954.1**	
Tegula fasciata	GQ160761.1	GQ160696.1	Belize
Tegula lividomaculata	**NC_029367**	**NC_029367**	
Omphalius rusticus	**MK170137.1**	**MK170137.1**	
Omphalius nigerrimus	**KX298895.1**	**KX298895.1**	
Omphalius pfeifferi	OL877094		South Korea
Turbo cornutus	**NC_061024.1**	**NC_061024.1**	**South Korea**
Turbo chrysostomus	**PQ276885.1**	**PQ276885.1**	**Malaysia**
Bolma rugosa	KT207824.1	KT207824.1	
Lunella granulata	**KX298890.1**	**KX298890.2**	
Lunella correensis	**MT185943.1**	**MT185943.1**	
Lunella cinerea	**PQ276881.1**	**PQ276881.1**	
Margarella antarctica	MN941904.1	MN941957.1	
Trochus sacellum	NC_067949	NC_067949	China
Umbonium thomasi	**NC_041307.1**	**NC_041307.2**	**South Korea**
Umbonium moniliferum	AB365227	AB505359.1	Japan
Umbonium giganteum	AB505314	AB505358.1	Japan
Astralium haematragum	**KX298891**	**KX298891**	
Angaria delphinus	KX298893.1	KX298893.1	
Angaria neglecta	**KR297248.1**	**KR297248.1**	
Rapana venosa	**NC_011193.1**	**NC_011193.1**	

Table S2. Pairwise genetic distances (%) among the superfamily Trochoidea based on partial mitochondrial COX1 gene sequences.

	NCBI Accession	Species	1	2	3	4	5	6	7	8	9	10	11	12	13	14	15	16	17	18	19	20	21	22	23	24	25	26	27	28	29	30	31	32	33	34	35	36
1	PV929813	*Rochia nilotica* Chuuk																																				
2	PX578333	*Rochia nilotica* Chuuk	0.00																																			
3	PX578334	*Rochia nilotica* Chuuk	0.00	0.00	0.00	0.00	0.00																															
4	PX578335	*Rochia nilotica* Chuuk	0.00	0.00	0.00	0.00	0.00																															
5	PX578336	*Rochia nilotica* Chuuk	0.00	0.00	0.00	0.00	0.00																															
6	PX578337	*Rochia nilotica* Chuuk	0.00	0.00	0.00	0.00	0.00																															
7	MK934681.1	*Rochia nilotica French Polynesia*	8.21	8.21	8.21	8.21	8.21	8.21																														
8	KT149313.1	*Rochia nilotica French Polynesia 1*	7.10	7.10	7.10	7.10	7.10	7.10	0.54																													
9	EU530151.1	*Rochia nilotica New Caledonia*	6.92	6.92	6.92	6.92	6.92	6.92	0.16	0.33																												
10	EU530150.1	*Rochia maxima Japan*	9.57	9.57	9.57	9.57	9.57	9.57	12.88	12.99	12.61																											
11	HE800628	*Tectus pyramis*	23.82	23.82	23.82	23.82	23.82	23.82	26.84	26.07	26.86	27.45																										
12	KY205709.1	*Rochia virgata Jordan*	18.62	18.62	18.62	18.62	18.62	18.62	22.28	22.77	21.90	19.97	24.33																									
13	NC_036068.1	*Tectus fenestratus*	26.07	26.07	26.07	26.07	26.07	26.07	26.20	26.81	26.01	28.10	16.80	29.17																								
14	EU530149	*Tectus tentorium*	25.72	25.72	25.72	25.72	25.72	25.72	26.39	27.10	25.96	25.99	19.36	27.97	10.07																							
15	EU530152	*Tegula brunnea*	24.97	24.97	24.97	24.97	24.97	24.97	25.05	24.90	24.81	24.57	24.32	25.20	21.97	23.75																						
16	NC_016954.1	*Tegula fasciata*	25.14	25.14	25.14	25.14	25.14	25.14	24.87	25.06	24.59	27.17	23.42	28.02	23.48	26.28	16.57																					
17	GQ160761.1	*Tegula lividomaculata*	25.33	25.33	25.33	25.33	25.33	25.33	26.05	26.21	25.64	24.56	25.41	23.17	21.19	23.41	1706	16.74																				
18	NC_029367	*Omphalius rusticus*	22.69	22.69	22.69	22.69	22.69	22.69	24.23	24.25	2404	21.53	24.83	24.44	23.63	24.94	11.59	15.94	16.40																			
19	MK170137.1	*Omphalius nigerrimus*	23.00	23.00	23.00	23.00	23.00	23.00	24.05	24.06	23.87	22.93	24.38	24.57	22.74	24.54	12.04	15.92	16.27	5.92																		
20	KX298895.1	*Omphalius pfeifferi*	24.33	24.33	24.33	24.33	24.33	24.33	24.92	24.97	24.92	23.03	24.51	24.37	21.89	23.44	12.58	18.03	15.95	4.85	6.55																	
21	OL877094	*Trochus sacellum*	29.67	29.67	29.67	29.67	29.67	29.67	29.04	31.22	30.78	29.78	29.27	32.56	26.40	30.70	30.76	32.08	29.01	30.22	30.95	30.09																
22	NC_061024.1	*Umbonium thomasi*	28.68	28.68	28.68	28.68	28.68	28.68	27.32	28.79	27.82	26.51	27.95	29.80	27.87	29.01	28.71	29.70	27.42	28.02	27.85	26.23	24.67															
23	PQ276885.1	*Umbonium moniliferum*	31.04	31.04	31.04	31.04	31.04	31.04	29.98	31.21	30.51	31.76	28.24	33.86	28.22	27.66	29.64	33.03	28.04	31.00	31.93	29.92	27.71	18.46														
24	KT207824.1	*Umbonium giganteum*	30.80	30.80	30.80	30.80	30.80	30.80	30.72	30.61	30.98	31.60	30.72	32.02	27.47	30.80	27.43	29.90	27.39	26.84	27.76	26.79	25.32	16.81	16.53													
25	KX298890.1	*Turbo cornutus*	19.10	19.10	19.10	19.10	19.10	19.10	20.83	21.68	21.18	21.18	20.96	21.02	22.10	23.31	23.19	22.04	22.26	21.19	20.49	21.56	31.83	27.24	32.39	28.85												
26	MT185943.1	*Turbo chrysostomus*	19.88	19.88	19.88	19.88	19.88	19.88	22.29	23.03	22.66	22.73	21.58	23.77	21.05	23.88	21.70	24.33	21.96	20.19	20.42	20.32	26.95	27.52	32.66	30.76	12.24											
27	PQ276881.1	*Bolma rugosa*	21.99	21.99	21.99	21.99	21.99	21.99	23.45	25.27	24.42	23.11	22.27	23.34	22.66	24.89	22.75	21.90	22.75	21.65	21.48	21.83	28.17	27.22	30.35	28.81	14.07	16.38										
28	MN941904.1	*Lunella granulata*	22.23	22.23	22.23	22.23	22.23	22.23	27.53	27.48	27.68	27.98	22.14	23.68	22.83	25.01	23.32	27.65	25.07	23.44	22.97	24.56	30.66	28.09	28.77	26.74	18.20	17.29	18.51									
29	NC_067949	*Lunella correensis*	21.70	21.70	21.70	21.70	21.70	21.70	27.50	27.66	28.10	25.92	23.75	23.10	23.28	27.24	23.15	26.67	23.79	23.59	22.53	22.56	31.20	29.21	29.05	29.49	18.62	17.20	19.56	9.54								
30	NC_041307.1	*Lunella cinerea*	21.81	21.81	21.81	21.81	21.81	21.81	25.25	26.21	25.53	23.83	20.78	21.94	22.88	23.64	23.85	24.86	23.83	23.71	23.23	25.60	31.10	28.80	33.90	31.66	17.25	17.66	16.93	15.22	15.79							
31	AB365227	*Margarella antarctica*	42.71	42.71	42.71	42.71	42.71	42.71	42.19	43.99	41.98	38.73	35.94	39.65	36.58	37.82	40.68	43.53	39.45	41.79	38.92	39.52	36.38	37.79	36.00	38.90	42.14	43.09	42.13	43.64	42.53	43.44						
32	AB505314	*Rapana venosa*	28.07	28.07	28.07	28.07	28.07	28.07	29.95	31.97	31.14	28.65	26.50	29.15	28.54	30.35	28.79	27.84	26.95	27.28	27.46	30.71	29.23	30.45	30.27	30.64	25.05	24.50	25.53	26.65	28.86	27.27	39.18					
33	KX298891	*Rochia conus Panglao-Philippines*	20.68	20.68	20.68	20.68	20.68	20.68	19.81	20.21	19.74	19.99	24.18	21.60	21.97	22.59	23.92	25.98	26.66	25.67	26.60	26.84	28.18	27.47	30.37	31.13	22.80	23.53	23.17	24.73	28.40	29.24	35.37	26.57				
34	KX298893.1	*Astralium haematragum*	23.51	23.51	23.51	23.51	23.51	23.51	26.30	26.07	26.34	26.23	24.44	24.49	25.20	26.65	22.96	23.65	23.91	23.44	23.27	24.39	29.71	29.55	34.22	30.63	18.32	19.82	19.63	21.05	22.06	20.76	42.75	29.90	25.18			
35	KR297248.1	*Angaria delphinus*	26.87	26.87	26.87	26.87	26.87	26.87	28.16	28.75	27.58	28.93	27.97	28.50	22.93	25.46	28.87	25.61	29.58	28.13	28.20	27.00	35.73	33.76	29.61	31.77	24.97	24.73	27.25	24.49	24.81	23.75	41.13	27.91	27.20	28.18		
36	NC_011193.1	*Angaria neglecta*	27.53	27.53	27.53	27.53	27.53	27.53	29.89	31.06	29.99	28.80	29.09	27.40	24.09	23.52	29.40	30.74	30.17	28.00	28.76	25.36	34.88	32.91	32.22	32.33	25.46	25.71	27.47	25.50	25.65	23.82	39.92	28.72	26.21	28.09	11.33	

Table S3. Pairwise genetic distances (%) among the superfamily Trochoidea based on partial mitochondrial 16S rRNA gene sequences.

Num-ber	NCBI Accession	Species	1	2	3	4	5	6	7	8	9	10	11	12	13	14	15	16	17	18	19	20	21	22	23	24	25	26	27	28	29	30	31	32	33
1	PV929813	*Rochia nilotica* Chuuk																																	
2	PX578451	*Rochia nilotica* Chuuk	0.00																																
3	PX578452	*Rochia nilotica* Chuuk	0.00	0.00																															
4	PX578453	*Rochia nilotica* Chuuk	0.00	0.00	0.00																														
5	PX578454	*Rochia nilotica* Chuuk	0.00	0.00	0.00	0.00																													
6	PX578455	*Rochia nilotica* Chuuk	0.00	0.00	0.00	0.00	0.00																												
7	HE800772.1	*R.nilotica* New Caledonia	3.15	3.15	3.15	3.15	3.15	3.15	3.15																										
8	HE800771.1	*Rochia maxima* Japan	4.82	4.82	4.82	4.82	4.82	4.82	4.82	6.03																									
9	HE800769.1	*Rochia conus*	15.58	15.58	15.58	15.58	15.58	15.58	15.58	15.97	14.71																								
10	KY205709.1	*Rochia virgate*	28.16	28.16	28.16	28.16	28.16	28.16	28.16	18.39	17.29	15.91																							
11	NC_036068.1	*Tectus pyramis*	34.71	34.71	34.71	34.71	34.71	34.71	34.71	19.02	18.29	17.50	34.80																						
12	HE800770.1	*Tectus fenestratus*	19.71	19.71	19.71	19.71	19.71	19.71	19.71	20.62	20.24	18.76	20.10	13.75																					
13	HE800773.1	*Tectus tentorium*	20.26	20.26	20.26	20.26	20.26	20.26	20.26	19.98	19.78	18.76	18.83	13.32	4.87																				
14	NC_016954.1	*Tegula brunnea*	39.71	39.71	39.71	39.71	39.71	39.71	39.71	25.54	25.85	25.27	40.78	40.77	23.64	22.61																			
15	GQ160696.1	*Tegula fasciata*	24.86	24.86	24.86	24.86	24.86	24.86	24.86	26.37	25.28	23.24	23.62	19.85	23.11	22.51	13.00																		
16	NC_029367	*Tegula lividomaculata*	37.91	37.91	37.91	37.91	37.91	37.91	37.91	26.83	25.23	21.06	38.59	41.00	22.99	21.17	25.59	9.94																	
17	MK170137.1	*Omphalius rusticus*	38.78	38.78	38.78	38.78	38.78	38.78	38.78	25.74	25.53	24.37	40.18	40.18	23.46	23.43	11.57	12.85	24.15																
18	KX298895.1	*Omphalius nigerrimus*	39.15	39.15	39.15	39.15	39.15	39.15	39.15	25.74	25.02	24.89	41.51	41.16	23.71	23.68	11.23	13.51	25.00	4.32															
19	NC_061024.1	*Turbo cornutus*	36.45	36.45	36.45	36.45	36.45	36.45	36.45	22.57	21.84	21.16	37.78	37.80	22.83	22.10	35.18	23.21	32.67	34.44	34.27														
20	PQ276885.1	*Turbo chrysostomus*	38.48	38.48	38.48	38.48	38.48	38.48	38.48	24.66	23.70	25.18	37.31	38.29	24.29	23.87	35.64	22.90	34.00	33.34	34.09	18.70													
21	KT207824.1	*Bolma rugosa*	40.44	40.44	40.44	40.44	40.44	40.44	40.44	22.95	22.06	19.70	39.91	39.16	21.78	21.58	37.17	18.09	33.56	36.91	38.57	28.49	28.56												
22	KX298890.2	*Lunella granulate*	37.08	37.08	37.08	37.08	37.08	37.08	37.08	21.97	22.29	23.64	37.38	40.79	24.12	22.69	36.31	22.61	34.23	36.49	37.80	26.40	26.64	32.31											
23	MT185943.1	*Lunella correensis*	38.08	38.08	38.08	38.08	38.08	38.08	38.08	22.20	23.25	22.91	38.56	41.73	23.08	21.92	34.85	23.32	34.76	35.87	37.29	25.72	27.64	32.28	6.22										
24	PQ276881.1	*Lunella cinereal*	42.63	42.63	42.63	42.63	42.63	42.63	42.63	25.69	26.71	24.65	42.74	43.19	24.45	23.54	39.00	22.91	37.41	39.67	39.81	30.69	33.59	35.59	28.96	26.99									
25	MN941957.1	*Margarella Antarctica*	23.41	23.41	23.41	23.41	23.41	23.41	23.41	24.40	24.68	22.13	21.50	21.09	21.29	21.87	25.97	24.72	24.25	26.11	26.88	22.09	22.51	19.01	24.83	24.78	22.53								
26	NC_067949	*Trochus sacellum*	44.06	44.06	44.06	44.06	44.06	44.06	44.06	29.25	28.66	26.84	43.09	45.57	25.75	24.75	43.86	27.74	42.52	43.16	43.63	42.62	41.09	44.33	41.02	41.86	45.76	24.21							
27	NC_041307.2	*Umbonium thomasi*	40.05	40.05	40.05	40.05	40.05	40.05	40.05	27.02	26.91	25.89	41.13	40.97	26.27	26.50	43.39	29.03	39.63	39.91	41.42	37.71	37.60	40.29	39.68	40.15	41.89	22.02	35.56						
28	AB505359.1	*Umbonium moniliferum*	26.52	26.52	26.52	26.52	26.52	26.52	26.52	27.87	27.52	26.81	30.25	24.30	26.43	25.22	26.84	27.65	26.66	27.90	28.16	26.47	26.49	26.21	24.11	24.14	28.24	21.78	22.96	11.00					
29	AB505358.1	*Umbonium giganteum*	25.72	25.72	25.72	25.72	25.72	25.72	25.72	27.64	26.72	25.46	29.06	24.53	26.98	26.00	26.79	28.54	27.54	27.40	28.74	26.70	26.47	25.25	24.09	24.37	27.68	22.64	20.77	9.97	5.54				
30	KX298891	*Astralium haematragum*	41.61	41.61	41.61	41.61	41.61	41.61	41.61	24.53	23.76	23.49	39.59	41.60	23.49	22.55	40.54	22.38	39.22	40.16	40.35	28.77	31.28	32.94	33.59	34.47	36.71	25.91	42.74	38.70	29.39	26.70			
31	KX298893.1	*Angaria Delphinus*	43.93	43.93	43.93	43.93	43.93	43.93	43.93	26.70	25.80	28.60	45.19	47.60	27.65	27.03	42.09	28.59	42.93	41.95	42.52	38.74	42.19	44.17	40.98	42.05	45.44	29.84	47.60	44.12	28.77	29.93	46.40		
32	KR297248.1	*Angaria neglecta*	43.32	43.32	43.32	43.32	43.32	43.32	43.32	26.28	25.40	27.80	44.32	46.62	26.68	26.13	41.60	29.24	43.30	41.72	42.89	39.27	41.12	43.94	41.13	41.13	44.91	30.58	47.67	43.33	29.41	30.64	47.08	14.00	
33	NC_011193.1	*Rapana venosa*	51.45	51.45	51.45	51.45	51.45	51.45	51.45	32.56	33.45	31.79	50.23	50.21	30.74	31.36	51.57	35.26	50.69	50.72	51.42	43.96	44.43	48.64	44.79	45.26	48.33	28.64	55.48	47.62	32.69	33.53	48.48	52.78	53.17
